# Metal stent versus plastic stent in endoscopic ultrasound‐guided hepaticogastrostomy for unresectable malignant biliary obstruction: Large single‐center retrospective comparative study

**DOI:** 10.1111/den.14956

**Published:** 2024-11-15

**Authors:** Daiki Yamashige, Susumu Hijioka, Yoshikuni Nagashio, Yuta Maruki, Yasuhiro Komori, Masaru Kuwada, Soma Fukuda, Shin Yagi, Kohei Okamoto, Daiki Agarie, Mark Chatto, Chigusa Morizane, Hideki Ueno, Shunsuke Sugawara, Miyuki Sone, Yutaka Saito, Takuji Okusaka

**Affiliations:** ^1^ Department of Hepatobiliary and Pancreatic Oncology National Cancer Center Hospital Tokyo Japan; ^2^ Department of Diagnostic Radiology National Cancer Center Hospital Tokyo Japan; ^3^ Endoscopy Division National Cancer Center Hospital Tokyo Japan; ^4^ Section of Gastroenterology, Department of Medicine Makati Medical Center Manila Philippines

**Keywords:** hepaticogastrostomy, interventional EUS, safety, stent

## Abstract

**Objective:**

Whether metal stents (MS) or plastic stents (PS) yield better outcomes for malignant biliary obstruction in endoscopic ultrasound‐guided hepaticogastrostomy (EUS‐HGS) is controversial. We aimed to compare outcomes of initial EUS‐HGS performed with MS or PS.

**Method**s**:**

In this single‐center retrospective study, we included patients (MS/PS groups: *n* = 151/72) with unresectable malignant biliary obstruction and performed multivariable analysis. The landmark date was defined as day 100 and used to evaluate the time to recurrent biliary obstruction (TRBO).

**Results:**

The clinical success rate was similar in both groups. The mean total bilirubin percentage decrease at week 2 was significantly higher in the MS group than in the PS group (−45.1% vs. −23.7%, *P* = 0.016). Median TRBO was significantly different between the MS and PS groups (183 and 92 days, respectively; *P* = 0.017). TRBO within 100 days was comparable in both groups but was significantly shorter only after 100 days in the PS group (adjusted hazard ratio 12.8, *P* < 0.001). Adverse events were significantly more common in the MS group (23.8% vs. 9.7%, *P* = 0.012), although they occurred relatively frequently even with PS in the cholangitis subgroup (*P*_interaction_ = 0.034). After endoscopic re‐intervention, TRBO tended to be longer with revision PS (hazard ratio 0.40, *P* = 0.47).

**Conclusions:**

Although MS provided early improvement of jaundice and long stent patency, PS provided a better safety profile and comparable stent patency until 100 days. PS might also be an adequate and optimal palliation method in EUS‐HGS.

## INTRODUCTION

Transpapillary stenting using endoscopic retrograde cholangiopancreatography (ERCP) is the standard treatment for malignant biliary obstruction (MBO).^1^ However, ERCP sometimes fails for several reasons. Recently, endoscopic ultrasound (EUS)‐guided biliary drainage (EUS‐BD), including EUS‐guided hepaticogastrostomy (EUS‐HGS), has been established as a salvage method.^2^, ^3^, ^4^ EUS‐BD is superior to percutaneous transhepatic biliary drainage, the conventional alternative drainage method, in effectiveness and safety profile.^5^, ^6^, ^7^ However, the incidence of adverse events (AEs) in EUS‐HGS remains high at 20–30%.^8^, ^9^, ^10^, ^11^, ^12^ A meta‐analysis demonstrated that EUS‐HGS had the highest AE rate among EUS‐BD approaches.^11^

It remains controversial whether metal stents (MS) or plastic stents (PS) yield better outcomes in EUS‐HGS. Covered self‐expandable MS (CSEMS) are a better option owing to their superior stent patency and prevention of bile leakage.^13^, ^14^ However, stent migration owing to stent shortening or early cholangitis and liver abscess caused by direct occlusion because of stent deployment has occasionally occurred with CSEMS.^15^, ^16^ Conversely, few reports have described PS use in EUS‐HGS.^17^, ^18^ Theoretically, PS are considered to be safer than MS because the lack of stent shortening reduces stent migration risk and the small diameter reduces the risk of incidental occlusion of the bile duct branches; however, this has not been conclusively proven to date.

In transpapillary biliary stenting, the stent patency of PS is inferior to that of MS because of its small diameter and lack of radial force.^19^ Because EUS‐HGS does not involve traversing malignant biliary strictures, the outcomes of MS and PS might theoretically differ between EUS‐HGS and transpapillary biliary stent placement. Limited data are available on the comparative outcomes of MS and PS.^20^, ^21^ Therefore, we aimed to compare clinical outcomes between MS and PS in EUS‐HGS for MBO.

## METHODS

### Study patients

We retrospectively included consecutive patients in whom initial EUS‐BD was attempted for unresectable MBO between March 2018 and January 2024 at a single center (National Cancer Center Hospital, Tokyo, Japan). Patients in whom initial EUS‐HGS was attempted for unresectable MBO for malignant gastroduodenal stenosis, surgically altered anatomy, endoscopic biliary drainage failure, or other reasons were included. Patients who underwent EUS‐guided choledochoduodenostomy, combined with stenting through the percutaneous transhepatic biliary drainage route, forward‐viewing EUS‐guided biliary drainage, antegrade stenting, or EUS‐guided hepaticoduodenostomy were excluded. This study was approved by the institutional review board of the National Cancer Center Hospital (approval no. 2018–149), and all patients provided written informed consent.

### Procedure details (EUS‐HGS)

A linear echoendoscope (GF‐UCT260 and GF‐UCT240; Olympus, Tokyo, Japan; EG‐580UT; FUJIFILM Medical, Tokyo, Japan) was used under moderate sedation. The B2 or B3 bile duct was punctured using a 19G fine needle aspiration (FNA) needle (EZ Shot 3 plus; Olympus Medical, Tokyo, Japan). The intrahepatic bile duct (IHBD) was confirmed by fluoroscopy, followed by the placement of a 0.025 inch guidewire. In some cases, puncture was performed using a 22G FNA needle (EZ Shot 3 plus; Olympus Medical), and a 0.018 inch guidewire was placed. Subsequently, the fistula was dilated using a bougie dilator (ES Dilator; ZEON Medical, Tokyo, Japan), balloon (REN; KANEKA, Osaka, Japan), drill (Tornus ES; Asahi Intec, Aichi, Japan), and/or an electric cautery dilator (Fine 025; Medico's Hirata, Osaka, Japan). Regarding the dilation strategy, before stent placement, we initially used a bougie but have shifted to using a drill dilator in recent years. If the stent delivery system could not pass through the fistula, additional dilation with a balloon dilator was performed. A cautery dilator was used if passage remained difficult. Subsequently, CSEMS or PS were deployed from the left IHBD to the stomach. The following 6 or 8 mm diameter CSEMS were used: X‐Suit NIR (laser‐cut type, 8 mm diameter; Olympus), covered BileRush Advance (laser‐cut type, 8 mm diameter; Piolax, Kanagawa, Japan), and EGIS Biliary Stent (braided type, 6 mm diameter; Sumitomo Bakelite, Tokyo, Japan). The following 7F PS were used: ERB‐USRT‐70‐150‐200‐SH5‐MK‐025 (straight type; Silux, Saitama, Japan) or Through & Pass Type‐IT (single pigtail type; Gadelius Medical, Tokyo, Japan).^17^ The stent was selected based on the time of purchase. CSEMS was mainly used in the early period (2018–2022), while PS was mainly used in the late period (2022–present). In this study, we did not perform scheduled stent replacement.

### Definitions

An expert hand was defined as one who had performed ≥30 EUS‐HGS procedures, as previously described.^22^ Technical success was defined as the deployment of MS or PS between the IHBD and stomach in the appropriate position. Technical success of endoscopic re‐intervention (ERI) was defined as a successful stent exchange and/or additional placement for initial EUS‐HGS route. Clinical success was defined as the rate of reduction in the serum total bilirubin (T‐Bil) level by 50% or to <2 mg/dL within 2 weeks. Time to recurrent biliary obstruction (TRBO) was defined as the time between stent deployment and the occurrence of cholangitis and jaundice, stent revision, or other biliary interventions because of recurrent biliary obstruction (RBO). The RBO etiology was determined based on computed tomography images taken prior to re‐intervention and the fluoroscopic and endoscopic images obtained during re‐intervention. Procedure‐related AEs were defined as events other than RBO.^23^ Among AEs, cholangitis was defined as the presence of fever and laboratory evidence of an inflammatory response and abnormal liver function relative to baseline data. This included the transient cholangitis after the procedure, nonobstructive cholangitis, or cholangitis caused by direct obstruction of the left bile duct owing to an EUS‐HGS‐related stent, other than RBO. Peritonitis was diagnosed based on the presence of clinical symptoms of peritoneal inflammation and the corresponding fluid collection due to bile leakage on computed tomography. Bleeding was defined as a reduction in serum hemoglobin levels by >2.0 g/dL. AEs were divided into early (occurring within 14 days) and late (occurring >14 days) AEs. Moreover, AEs were graded according to the American Society for Gastrointestinal Endoscopy lexicon.^24^

### Statistical analysis

Continuous variables are presented as medians (interquartile ranges), and categorical variables are presented as numbers (percentages). Qualitative and quantitative differences between groups were evaluated using the χ^2^‐test or Fisher's exact test and the Mann–Whitney *U*‐test, Kruskal–Wallis test, or unpaired *t*‐test. TRBO and overall survival (OS) were calculated using Kaplan–Meier analysis and compared using the log‐rank test. Patients with the following events were censored in the analysis of TRBO: stent removal without RBO, loss to follow‐up, or death before RBO. The landmark date was defined as 100 days and was used to evaluate TRBO. In the analysis of RBO until the 100‐day landmark, patients who had been observed for >100 days were censored at the 100‐day time point. In the analysis after the 100‐day landmark, only patients who were observed for >100 days were analyzed. For adjusting the time gap, treatment periods were classified into two groups: 2018–2020 and 2021–present. Additionally, Cox hazard regression and binary logistic regression were used to calculate hazard ratios (HRs) and odds ratios (ORs) with 95% confidence intervals (CIs) in the overall and subgroup analyses. All reported *P*‐values were two‐sided, and a *P*‐value <0.05 was considered statistically significant. All statistical analyses were performed using SPSS version 27.0 (IBM Corp., Armonk, NY, USA).

## RESULTS

### Patients

After excluding four cases of unsuccessful puncture (all technical failure cases) because of poor bile duct dilation, a total of 223 patients (MS group: 151; PS group: 72) who underwent initial EUS‐HGS for MBO were included (Fig. S1). The baseline characteristics are shown in Table 1.

**Table 1:** Baseline characteristics

Baseline characteristic	All patients; *n* = 223	MS group; *n* = 151	PS group; *n* = 72	*P*‐value
Age, years	68 (58–75)	67 (57.5–74.5)	69.5 (59.5–76.5)	0.157
Sex (male)	115 (51.6)	79 (52.3)	36 (50.0)	0.746
Performance status, 0/1/2/3/4	47/141/30/5/0	35/91/21/4/0	12/50/9/1/0	0.563
Primary disease	–	–	–	0.082
Pancreatic cancer	108 (48.4)	73 (48.3)	35 (48.6)	0.970
Biliary tract cancer	54 (24.2)	30 (19.9)	24 (33.3)	0.028
Other cancers	61 (27.4)	48 (31.8)	13 (18.1)	0.031
Disease stage status	–	–	–	0.413
Local invasion	49 (22.0)	30 (19.9)	19 (26.4)	0.271
Metastasis	125 (56.1)	89 (58.9)	36 (50.0)	0.208
Recurrence	49 (22.0)	32 (21.2)	17 (23.6%)	0.683
Stenosis status				
Distal stenosis	165 (74.0)	116 (76.8)	49 (68.1)	0.163
Hilar stenosis	58 (26.0)	35 (23.2)	23 (31.9)	–
Cholangitis status	49 (22.0)	36 (23.8)	13 (18.1)	0.329
Ascites	46 (20.6)	31 (20.5)	15 (20.8)	0.958
Re‐intervention after ERCP	61 (27.4)	38 (25.2)	23 (31.9)	0.288
After metal stent deployed^†^	45 (20.2)	29 (19.2)	16 (22.2)	0.600
After plastic stent deployed^†^	18 (8.1)	12 (7.9)	6 (8.3)	0.921
Previous duodenal stent deployed	58 (26.0)	37 (24.5)	21 (29.2%)	0.458
Surgical anatomy	55 (24.7)	36 (23.8)	19 (26.4)	0.680
Baseline data				
Total bilirubin, mg/dL	2.6 (0.9–5.8)	2.8 (1.0–6.0)	2.5 (0.8–5.5)	0.288
Aspartate aminotransferase, U/L	80 (41.5–137)	89 (44.5–137)	80 (38–133)	0.285
Alanine transaminase, U/L	78 (40–140)	85.0 (42.5–165)	66 (38.5–104)	0.080
Concomitant chemotherapy	120 (53.8)	80 (53.0)	40 (55.6)	0.718
Follow‐up duration, days	157 (52–285)	161 (83–285)	156 (78–221)	0.835

Data are presented as *n* (%) or median (interquartile range).

ERCP, endoscopic retrograde cholangiopancreatography; MS, metal stent; PS, plastic stent.

^†^ Duplicated number.

Procedure details are presented in Table 2. Malignant gastroduodenal stenosis (60.9 vs. 52.8%, *P* = 0.25) was the most common reason for performing EUS‐HGS in the MS and PS groups, with no significant difference in any of the reasons. In fistula dilation methods, bougie dilators were used more frequently in the MS group, whereas drill dilators were used more frequently in the PS group.

**Table 2:** Procedure details

Variable	All patients; *n* = 223	MS group; *n* = 151	PS group; *n* = 72	*P*‐value
Reason	–	–	–	0.669
Gastric outlet obstruction	130 (58.3)	92 (60.9)	38 (52.8)	0.248
Altered anatomy	46 (20.6)	28 (18.5)	18 (25.0)	0.265
Not completed or failed ERCP	21 (9.4%)	15 (9.9)	6 (8.3)	0.702
Hilar stenosis (ERCP not performed)	15 (6.7)	7 (4.6)	8 (11.1)	0.088
Others	11 (4.9)	9 (5.9%)	2 (2.8)	0.305
Technical level				
Expert hand	23 (10.3)	15 (9.9)	8 (11.1)	0.477
Nonexpert hand	200 (89.7)	136 (90.1)	64 (88.9)	–
Puncture site in EUS‐HGS	–	–	–	–
B2	63 (28.3)	46 (30.5)	17 (23.6)	0.288
B3	160 (71.7)	105 (69.5%)	55 (76.4)	–
Punctured bile duct diameter, mm	3.3 (2.5–5.0)	3.2 (2.8–5.0)	3.3 (2.3–4.9)	0.353
Puncture needle, 22G	16 (7.2)	9 (6.0)	7 (9.7)	0.309
Fistula dilation	197 (88.3)	134 (88.7)	63 (87.5)	0.787
Bougie dilator^†^	156 (70.0%)	126 (83.4)	30 (41.7)	<0.001
Balloon dilator^†^	31 (13.9)	20 (13.2)	11 (15.3)	0.682
Drill dilator^†^	35 (15.7)	4 (2.6)	31 (43.1)	<0.001
Cautery dilator^†^	1 (0.4)	1 (0.7)	0 (0.0)	0.677
Multiple dilation devices used	26 (11.7)	17 (11.3)	9 (12.5)	0.787
Stent type	–	–	–	–
Metal stent	–	–	–	–
Laser‐cut type	–	136 (90.1)	–	–
Braided type	–	15 (9.9)	–	–
Plastic stent	–	–	–	–
Straight type	–	–	54 (75.0)	–
Single pigtail type	–	–	18 (25.0)	–
Stent length	–	–	–	–
8/10/12/14/15 cm	129/9/17/14/54	129/9/13/0/0	0/0/4/14/54	<0.001
Stent diameter				
6 mm/8 mm/7F	18/133/72	18/133/0	0/0/72	<0.001
Simultaneous duodenal stenting	28 (12.6)	23 (15.2)	5 (6.9)	0.081
Procedure time, min	50 (36–73.5)	50 (34.5–70)	50.5 (40–80)	0.397
Treatment period				
2018–2020	109 (48.9%)	98 (64.9)	11 (15.3)	<0.001
2021–present	114 (51.1)	53 (35.1)	61 (84.7)	–

Data are presented as *n* (%) or median (interquartile range).

ERCP, endoscopic retrograde cholangiopancreatography; EUS‐HGS, endoscopic ultrasound‐guided hepaticogastrostomy; MS, metal stent; PS, plastic stent.

^†^ Duplicated number.

### Short‐term outcomes

**Figure 1:**
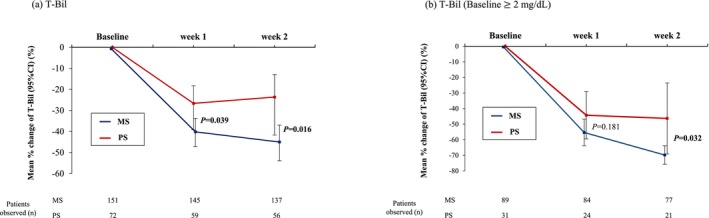
Short‐term improvement of total bilirubin (T‐Bil) level from baseline to weeks 1 and 2. Comparison of mean percentage change in T‐Bil levels from baseline to weeks 1 and 2 using unpaired *t*‐test. CI, confidence interval; MS, metal stent; PS, plastic stent.

The clinical success rates in the MS and PS groups were 87.4% and 83.3%, respectively, with no significant difference (*P* = 0.41) (Table 3). The mean percentage decreases in T‐Bil levels in the MS and PS groups were −40.2% vs. −26.6% at week 1 (*P* = 0.039) and −45.1% vs. −23.7% at week 2 (*P* = 0.016). This statistical trend was also observed in the cohort with jaundice (T‐Bil ≥2 mg/dL; *P* = 0.032) (Fig. 1). The mean percentage change in aspartate aminotransferase or alanine aminotransferase levels did not significantly differ (Fig. S2).

**Table 3:** Clinical outcomes

Variable	All patients; *n* = 223	MS group; *n* = 151	PS group; *n* = 72	*P*‐value
Clinical success	192 (86.1)	132 (87.4)	60 (83.3)	0.410
Total bilirubin value (at week 2), mg/dL	0.9 (0.6–1.4)	0.8 (0.6–1.45)	1.1 (0.5–1.6)	0.474
Aspartate aminotransferase value (at week 2), U/L	31.5 (21.5–58)	31 (22–48)	34 (20–72.5)	0.690
Alanine transaminase value (at week 2), U/L	27.5 (15–47.5)	28 (15–46)	25 (15.5–48.5)	0.773
Adverse event	43 (19.3)	36 (23.8)	7 (9.7)	0.012
Early (within 14 days)	37 (16.6)	32 (21.2)	5 (6.9)	0.007
Late (after 14 days)	6 (2.7)	4 (2.6)	2 (2.8%)	0.631
Grade (mild/moderate/severe)	7/29/7	6/24/6	1/5/1	0.970
Cholangitis	15 (6.7)	14 (9.3)	1 (1.4)	0.020
Liver abscess	9 (4.0)	8 (5.3)	1 (1.4)	0.153
Peritonitis (from bile leakage)	9 (4.0)	7 (4.6)	2 (2.8)	0.400
Bleeding	4 (1.8%)	3 (2.0%)	1 (1.4%)	0.611
Perforation (from stent migration)	4 (1.8)	2 (1.3)	2 (2.8)	0.389
Cholecystitis	2 (0.9)	2 (1.3)	0 (0.0)	0.327
RBO	98 (43.9)	61 (40.4)	37 (51.4)	0.122
Etiology of RBO				
Sludge	34 (15.2)	9 (6.0)	25 (34.7)	<0.001
Hyperplasia	22 (9.9%)	22 (14.6)	0 (0.0)	<0.001
New biliary stricture	13 (5.8)	8 (5.3)	5 (6.9)	0.415
Stent dislocation	12 (5.4)	9 (6.0)	3 (4.2)	0.420
Stent kinking	6 (2.7)	5 (3.3)	1 (1.4)	0.369
Development of stone	6 (2.7)	4 (2.6)	2 (2.8)	0.631
Hemobilia	5 (1.8)	4 (2.6)	1 (1.4)	0.480
Early RBO (≤100 days)	75 (33.6)	46 (30.5)	29 (40.3)	0.147
Late RBO (>100 days)	23 (10.3)	15 (9.9)	8 (11.1)	0.787

Data are presented as *n* (%) or median (interquartile range).

MS, metal stent; PS, plastic stent; RBO, recurrent biliary obstruction.

### Long‐term outcomes

**Figure 2:**
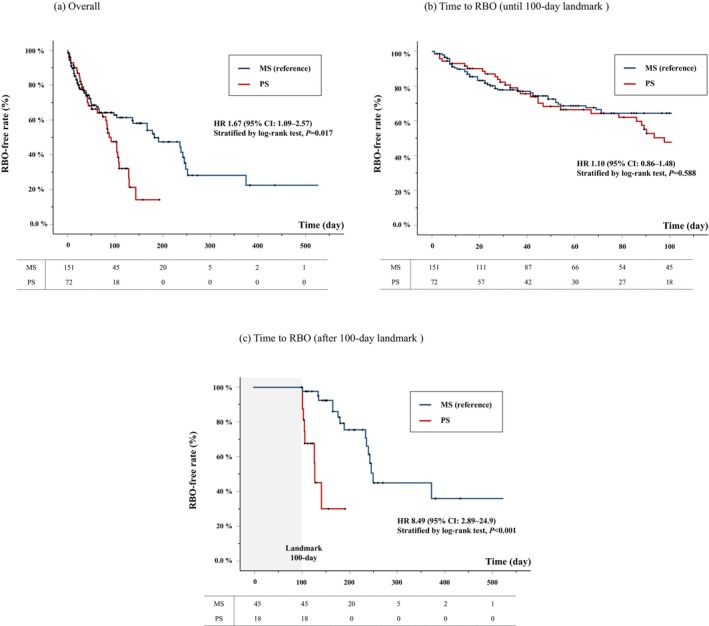
Time to recurrent biliary obstruction (RBO) in the metal stent vs. plastic stent. Kaplan–Meier curve of time to RBO. (a) Overall, (b) time to RBO (until the 100 day landmark), and (c) time to RBO (after the 100 day landmark). CI, confidence interval; HR, hazard ratio; MS, metal stent; PS, plastic stent.

During the observation period, RBO developed in 40.4% (61/151) and 51.4% (37/72) of patients in the MS and PS groups, respectively (Table 3). In the MS group, tissue hyperplasia was the most common RBO etiology. In the PS group, sludge was the most common RBO etiology. In early RBO, particularly in the MS group, hyperplasia, stent dislocation, and stent kinking were relatively common (Table S1). The median TRBO values were 183 days (95% CI 102–264) and 92 days (95% CI 71.1–113) in the MS and PS groups, respectively (HR 1.67 [95% CI 1.09–2.57], *P* = 0.017) (Fig. 2a). In the multivariate analysis, TRBO did not differ significantly between two groups (adjusted hazard ratio [aHR]: 1.53 [95% CI 0.66–3.52], *P* = 0.32) (Table 4).

**Table 4:** Factors associated with recurrent biliary obstruction in multivariate Cox regression analysis

	Overall (*n* = 223)			
Variable	Univariate; HR (95% CI)	*P*‐value	Multivariate; aHR (95% CI)	*P*‐value
Age (per year)	1.01 (0.99–1.02)	0.519	1.02 (1.00–1.04)	0.091
Sex (male)	1.44 (0.97–2.18)	0.072	1.51 (0.97–2.37)	0.069
Performance status (≥2)	0.79 (0.41–1.52)	0.478	0.73 (0.35–1.54)	0.412
Primary disease				
Pancreatic cancer	0.79 (0.53–1.18)	0.248	0.91 (0.52–1.59)	0.740
Biliary tract cancer	1.08 (0.67–1.73)	0.756	0.84 (0.42–1.68)	0.619
Disease status				
Local invasion (reference)	1.0 (reference)	–	1.0 (reference)	–
Metastasis	2.14 (1.21–3.78)	0.009	2.37 (1.26–4.44)	0.007
Recurrence	1.77 (0.92–3.40)	0.087	1.78 (0.82–3.87)	0.144
Stenosis status				
Hilar stenosis (vs. distal stenosis)	1.06 (0.68–1.66)	0.786	1.07 (0.62–1.86)	0.807
Cholangitis status	1.06 (0.62–1.81)	0.846	1.03 (0.57–1.85)	0.923
Ascites	0.78 (0.42–1.43)	0.413	0.85 (0.44–1.62)	0.615
Re‐intervention after ERCP	0.97 (0.62–1.52)	0.885	1.22 (0.71–2.10)	0.480
Previous duodenal stent deployed	0.92 (0.58–1.45)	0.709	0.87 (0.51–1.48)	0.605
Baseline data				
Total bilirubin, mg/dL (per 1)	1.02 (0.98–1.06)	0.353	1.03 (0.98–1.08)	0.255
Aspartate aminotransferase, U/L) (per 1)	1.00 (1.00–1.00)	0.877	1.00 (1.00–1.00)	0.835
Alkaline phosphatase, U/L (per 1)	1.00 (1.00–1.00)	0.353	1.00 (1.00–1.00)	0.487
Stenting to B2 (vs. B3)	0.65 (0.40–1.07)	0.091	0.75 (0.43–1.30)	0.304
Fistula dilation (vs. non‐dilation)	0.99 (0.53–1.86)	0.985	1.18 (0.60–2.34)	0.628
Stent length (≥10 cm)	1.70 (1.12–2.59)	0.013	1.35 (0.62–2.95)	0.458
Simultaneous duodenal stenting	0.78 (0.39–1.55)	0.481	0.74 (0.35–1.54)	0.418
Plastic stent (vs. metal stent)	1.67 (1.09–2.57)	0.019	1.53 (0.66–3.52)	0.321
Treatment period: 2021–present (vs. 2018–2020)	1.27 (0.84–1.90)	0.256	1.01 (0.58–1.74)	0.976
Non‐expert hand (vs. expert hand)	1.10 (0.78–1.65)	0.652	1.08 (0.60–1.90)	0.758

aHR, adjusted hazard ratio; CI, confidence interval; ERCP, endoscopic retrograde cholangiopancreatography; HR, hazard ratio.

Until the 100‐day landmark, TRBO was similar in both groups (HR 1.10, 95% CI 0.86–1.48, *P* = 0.588) (Fig. 2b). After the 100 day landmark, 45 and 18 cases were compared between the MS and PS groups, respectively. TRBO after 100 days was significantly shorter in the PS group (HR 8.49, 95% CI 2.89–24.9, *P* < 0.001) (Fig. 2c). In the multivariate analysis, the PS group showed an independently shorter TRBO after 100 days (aHR 12.8, 95% CI 3.17–51.5, *P* < 0.001) (Table 5).

**Table 5:** Factors associated with recurrent biliary obstruction (RBO) (until/after the 100‐day landmark) in multivariate Cox regression analysis

Analysis cohort	Overall (*n* = 223)		Landmark analysis (*n* = 63)	
RBO	Until the 100‐day landmark (*n* = 75)		After the 100‐day landmark (*n* = 23)	
Variable	Multivariate; aHR (95% CI)	*P*‐value	Multivariate; aHR (95% CI)	*P*‐value
Primary disease				
Pancreatic cancer	0.95 (0.52–1.73)	0.859	–	–
Biliary tract cancer	0.88 (0.43–1.80)	0.723	–	–
Disease status				
Local invasion (reference)	1.0 (reference)	–	1.0 (reference)	–
Metastasis	1.81 (0.94–3.51)	0.078	6.77 (1.72–26.6)	0.006
Recurrence	1.63 (0.71–3.73)	0.250	7.73 (1.50–39.8)	0.015
Hilar stenosis (vs. distal stenosis)	0.99 (0.53–1.85)	0.986	0.99 (0.36–2.68)	0.978
Cholangitis status	1.08 (0.58–2.00)	0.809	0.87 (0.17–4.32)	0.861
Ascites	0.66 (0.32–1.37)	0.267	0.97 (0.26–3.61)	0.968
Re‐intervention after ERCP	1.08 (0.59–1.97)	0.816	2.63 (0.91–7.61)	0.074
Previous duodenal stent deployed	0.90 (0.50–1.62)	0.732	–	–
Total bilirubin, mg/day (per 1)	1.03 (0.98–1.08)	0.266	–	–
Stent length (≥10 cm)	1.28 (0.57–2.87)	0.543	–	–
Plastic stent (vs. metal stent)	1.16 (0.50–2.71)	0.728	12.8 (3.17–51.5)	<0.001
Treatment period: 2021–present (vs. 2018–2020)	0.99 (0.56–1.75)	0.963	0.53 (0.17–1.62)	0.265
Nonexpert hand (vs. expert hand)	1.12 (0.79–1.73)	0.853		

aHR, adjusted hazard ratio; CI, confidence interval; ERCP, endoscopic retrograde cholangiopancreatography.

**Figure 3:**
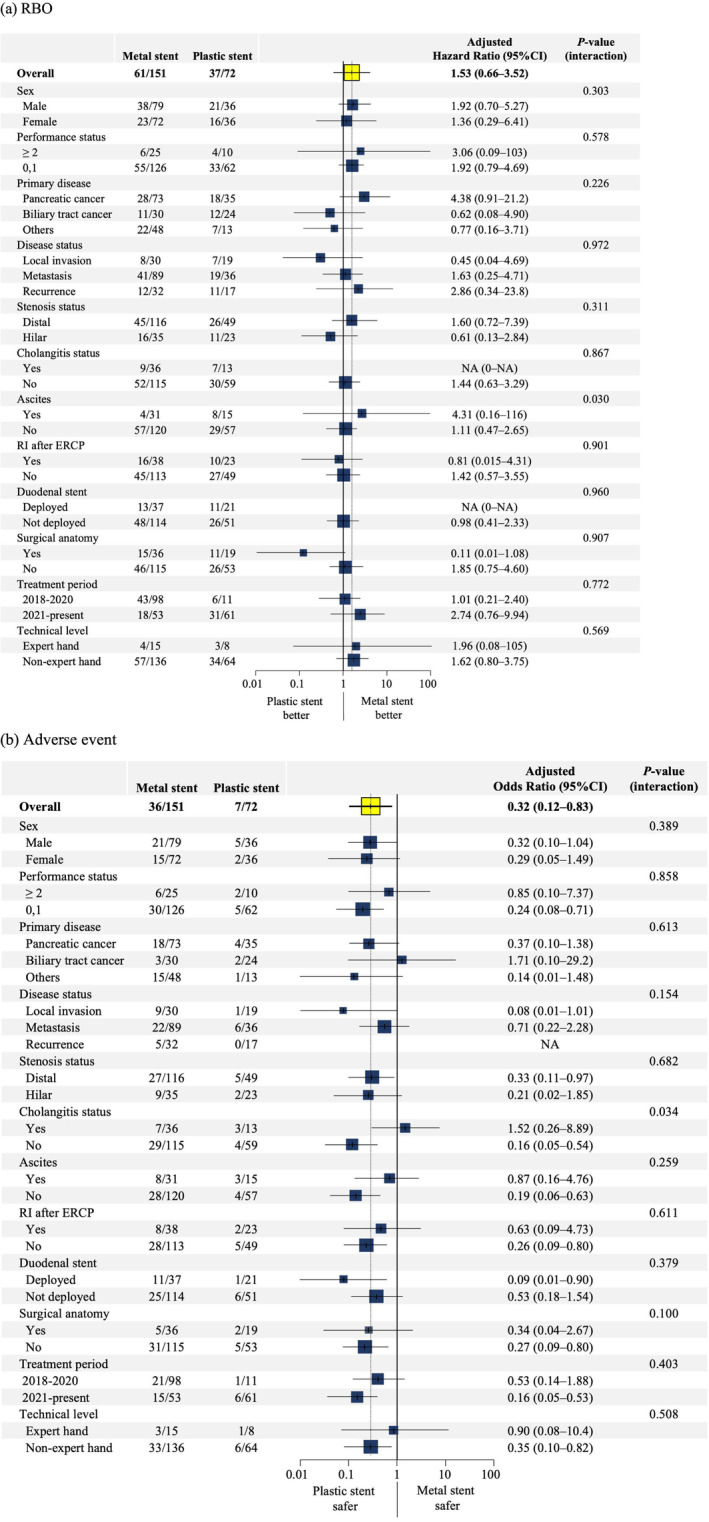
Forest plot of the treatment‐adjusted outcomes on (a) recurrent biliary obstruction (RBO) and (b) adverse events in subgroup analyses. Cox and logistic regression analyses were performed to estimate the adjusted hazard ratios and odds ratios with 95% confidence intervals (CIs) for the associations between metal and plastic stents. The position of each square represents the point estimate of the treatment effect, and the error bars represent 95% CIs. The size of the squares is proportional to the precision of the estimates. The multivariable analysis included the following adjustments: (a) primary disease type, disease status, stenosis status, cholangitis status, ascites, re‐intervention after endoscopic retrograde cholangiopancreatography (ERCP), previous duodenal stent deployed, total bilirubin (mg/dL, per 1), stent length, and timing 2021 (vs. 2018–2020); and (b) stenosis status, cholangitis status, ascites, fistula dilation, and timing 2021 (vs. 2018–2020).

Subgroup analysis results regarding RBO are shown in Figure 3a. The patients with ascites had better outcomes in the MS group (*P*_interaction_ = 0.030). In addition, there was no significant difference in OS between two groups (Fig. S3).

### Re‐intervention

Among the 98 patients who developed RBO, ERI was planned in 89 patients. The technical success rates of ERI were 87.7% (50/57) in the MS group and 96.9% (31/32) in the PS group (*P* = 0.14). In the MS group, many technical failure cases were due to the difficulty in passing through the stricture caused by hyperplasia (Table S2). On classifying the four groups from the first stent to the revision stent, the clinical success rate was significantly lower in the MS–MS group (cases in which the MS was placed as the initial and revision stents) at 66.7% (8/12) (*P* = 0.021) (Table S3). After ERI, TRBO tended to be longer in the group that used PS as revision stents, although the difference was not significant (HR 0.40, 95% CI 0.14–1.64, *P* = 0.47) (Fig. S4a). Additionally, regardless of which stent type was used first, TRBO tended to be longer when PS was used as revision stents (Fig. S4b).

### Safety profile

The total AE rate was significantly higher in the MS group than in the PS group (23.8% vs. 9.7%, *P* = 0.012). Early AEs were significantly more common in the MS group (*P* = 0.007) (Table 3). In the multivariate analysis, PS posed a significantly lower risk of AEs than MS (adjusted OR 0.32, 95% CI 0.12–0.83, *P* = 0.019) (Table S4). The adjusted risk in both groups in the subgroup analysis is shown in Figure 3b. Most subgroups tended to have a lower risk of AEs when PS was used. In the presence of cholangitis, however, AEs occurred relatively frequently even with PS (*P*_interaction_ = 0.034).

## DISCUSSION

Although a few reports had compared MS and PS in EUS‐HGS,^14^, ^20^, ^21^ the differences in characteristics beyond TRBO between the two stents remained unclarified. In this study, consistent with previous reports, the TRBO of PS was significantly shorter; however, rather than being inferior, PS was shown to be equivalent to MS for up to 100 days and was associated with fewer AEs and relatively favorable outcomes in ERI. Thus, to the best of our knowledge, we have demonstrated for the first time the advantages of PS in EUS‐HGS.

MS provides better long stent patency than PS in transpapillary stenting, and the TRBO of PS was reduced from the early time of stent deployment.^19^, ^25^, ^26^ Regarding EUS‐HGS, however, TRBO was similar for both stent types until 100 days. Here, these profile distinctions may indicate the difference in the peri‐stent environment, i.e., the presence or absence of a direct tumor impact on the stent. In contrast, in the 100 day landmark analysis, PS was an independent factor for RBO after 100 days, which may be associated with a smaller diameter and nonself‐expansion ability. Interestingly, these disadvantages affected RBO only after 100 days in EUS‐HGS.

Tissue hyperplasia was the most common etiology of RBO in the MS group.^21^, ^27^ As the EUS‐HGS stent is placed in the IHBD, the larger stent lumen and radial force cause tissue hyperplasia. Tissue hyperplasia can make it difficult to pass through the stenosis and deploy the revision stent. Therefore, the technical success rate of ERI after MS placement was relatively low. In the PS group, however, sludge was the most common cause of RBO. The technical success rate of ERI was high because the deployed PS was easily removed and replaced with another PS.^28^ Because few ductal changes in IHBD had occurred at the initial intervention and re‐intervention, the long‐term outcomes of PS as revision stents are thought to be similar to those of the initial PS. Contrastingly, after MS, it is necessary to place the revision stent in a more proximal position to surpass the stricture caused by hyperplasia. This results in an expanded area of sacrificed drainage, which we believe is a reason for the shorter TRBO. Nakai *et al*.^27^ also reported that using PS as a revision stent results in a slightly longer TRBO. However, data on ERI are limited,^29^ and further studies are needed.

CSEMS prevent bile leakage and tract bleeding,^12^, ^13^, ^17^ but the large diameter and radial force can sometimes cause cholangitis because of the accidental direct obstruction of the IHBD.^18^, ^30^ Consequently, AEs, particularly early AEs, were significantly more common in the MS group. However, the incidence of such AEs was lower in the PS group because a smaller diameter and lack of radial force reduce the risk of directly obstructing the IHBD. In addition, peritonitis (due to bile leakage), a concern in PS use, was low in our study, and was not a significant risk of PS use (0.0–4.7%).^18^, ^21^, ^28^ In addition, considering the technical expertise is also important. In this study, the procedure time was slightly longer, at 50 min. This may be attributed to the fact that 89.7% (200/223) of the procedures were performed by nonexpert hands with an experience of fewer than 30 cases of EUS‐HGS.^22^ However, it is intriguing that PS was shown to be independently safe regardless of the presence of an expert hand.

Regarding the stent selection options based on these results, PS might be the better choice for initial EUS‐HGS from the perspective of AEs. Optimally, the PS should be regularly replaced within 100 days, when the risk of RBO is low. Furthermore, adding antegrade stenting to PS, which has recently been shown to be effective, may be a rational strategy to compensate for the significantly lower stent patency beyond 100 days.^31^, ^32^ In cases in which prognosis is limited (the likelihood of ERI is low), MS placement may be an option because MS as a revision stent has shown a lower rate of AEs. Recently, the utility of dedicated partially CSEMS, developed to reduce adverse events, has been reported for MS, although further investigation is warranted.^27^

This study had some limitations. First, this study had a retrospective design, which has inherent limitations. To adjust for potential confounding factors, we used multivariate analyses. Second, in subgroup and re‐intervention analyses, the sample size of each cohort was relatively small, which may have limited the statistical power to detect significance and resulted in limited data. Third, the recent advancements in EUS‐HGS have introduced potential differences in treatment outcomes, including safety, depending on the treatment period. Therefore, to adjust for this bias as much as possible, we divided the total study sample into two groups by treatment period: 2018–2020 and 2021–present. Finally, the definitions, especially AEs, tend to be vague and subjective because of a single‐center nature. In the future, prospective, randomized controlled trials and large‐scale multicenter studies are warranted.

In conclusion, although MS provided early improvement of jaundice and long stent patency after 100 days, PS also provided a good safety profile and comparable stent patency until 100 days. Thus, PS might also be a safer option in EUS‐HGS for MBO. The best stent selection should be considered based on this evidence and patients' individual backgrounds.

## CONFLICT OF INTEREST

Authors declare no conflict of interest for this article.

## FUNDING INFORMATION

This work was supported by The National Cancer Center Research and Development Fund (grant number 2022‐A‐16).

## ETHICS STATEMENT

Approval of the research protocol by an Institutional Reviewer Board: This study was approved by the institutional review board of the National Cancer Center Hospital (approval no. 2018–149).

Informed Consent: All patients provided written informed consent for use of their data and the endoscopic procedures.

Registry and the Registration No. of the study/trial: N/A.

Animal Studies: N/A.

## Supporting information


**Figure S1** Study flowchart.
**Figure S2** Short‐term improvement of aspartate aminotransferase (AST) and alanine aminotransferase (ALT) levels from baseline to weeks 1 and 2.
**Figure S3** Overall survival in patients who underwent initial endoscopic ultrasound‐guided hepaticogastrostomy (EUS‐HGS).
**Figure S4** Time to recurrent biliary obstruction with a revision stent.

**Table S1** Etiology of recurrent biliary obstruction (RBO) until/after the 100 day landmark.
**Table S2** Technical failure cases of endoscopic re‐intervention.
**Table S3** Procedures and short‐term outcomes of endoscopic re‐intervention.
**Table S4** Adjusted relative risk of adverse events in stent types (metal stent vs. plastic stent).
