# Supplementary material for: Metal stent versus plastic stent in endoscopic ultrasound‐guided hepaticogastrostomy for unresectable malignant biliary obstruction: Large single‐center retrospective comparative study
Source: Dig Endosc. 2024 Nov 15;37(1):117–29. doi: 10.1111/den.14956 (PMC11718138; doi:10.1111/den.14956)
Supplement: Supplementary file 1 — Figure S1 Study flowchart. Figure S2 Short‐term improvement of aspartate aminotransferase (AST) and alanine aminotransferase (ALT) levels from baseline to weeks 1 and 2. Figure S3 Overall survival in patients who underwent initial endoscopic ultrasound‐guided hepaticogastrostomy (EUS‐HGS). Figure S4 Time to recurrent biliary obstruction with a revision stent. [file DEN-37-117-s001.pptx]

## Slide 1
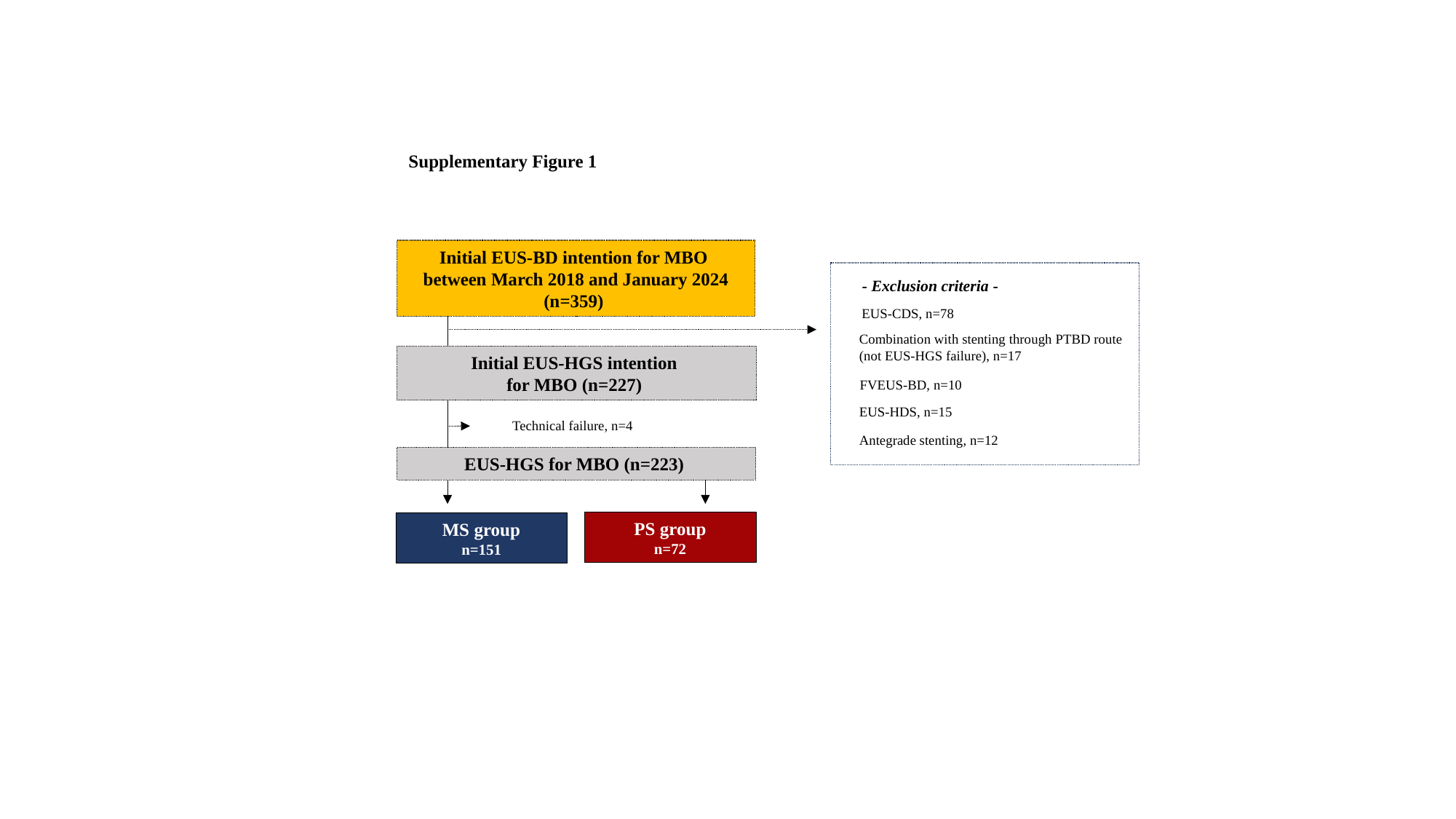

Supplementary Figure 1
Initial EUS-BD intention for MBO
between March 2018 and January 2024 (n=359)
- Exclusion criteria -
EUS-CDS, n=78
Combination with stenting through PTBD route
(not EUS-HGS failure), n=17
Initial EUS-HGS intention
for MBO (n=227)
FVEUS-BD, n=10
EUS-HDS, n=15
Technical failure, n=4
Antegrade stenting, n=12
EUS-HGS for MBO (n=223)
PS group
n=72
MS group
n=151

## Slide 2
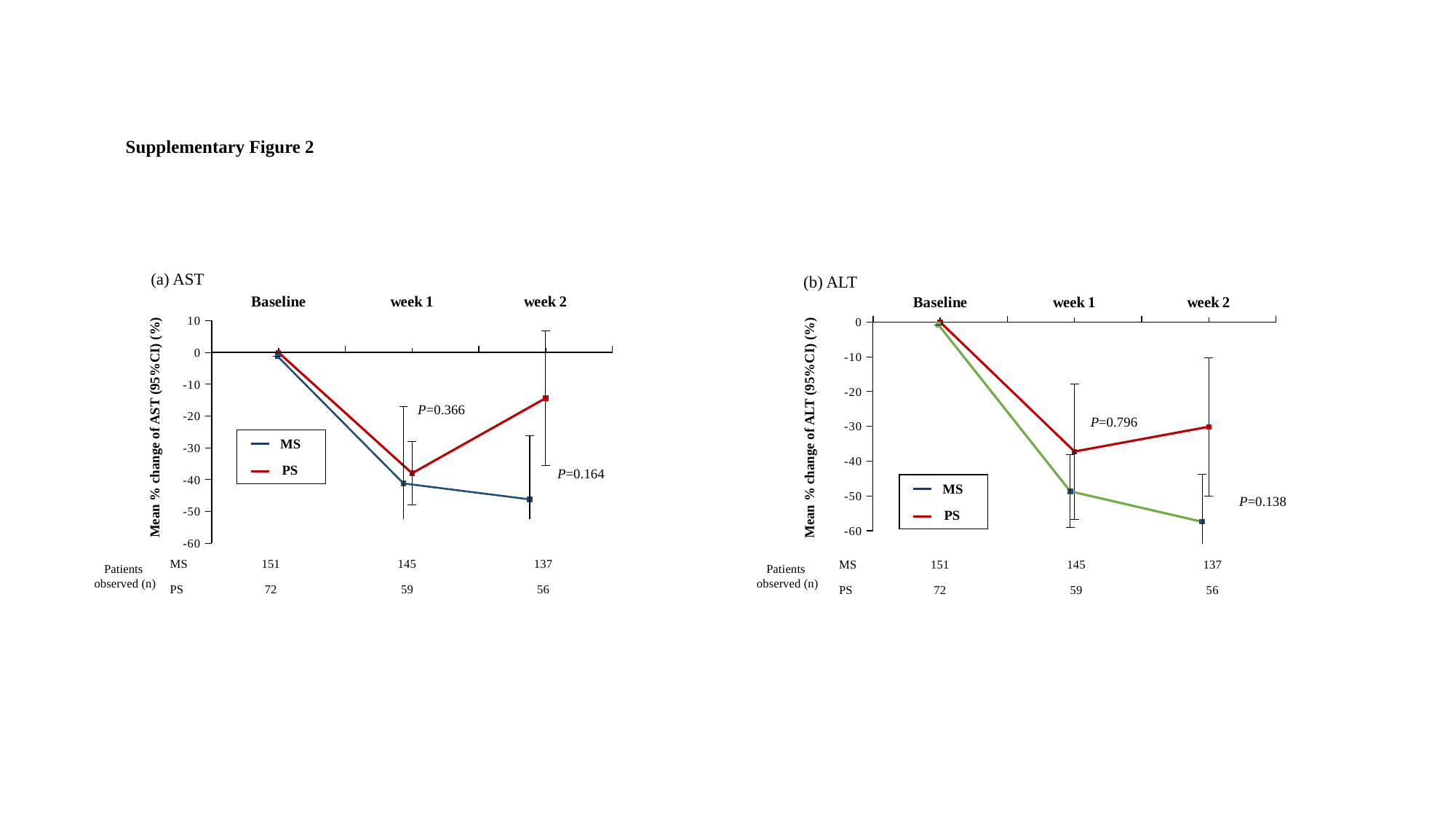

Supplementary Figure 2
(a) AST
### Chart
| Category | PS |
|---|---|
| Baseline | 0.0 |
| week 1 | -37.9703 |
| week 2 | -14.3468 |
### Chart
| Category | MS |
|---|---|
| Baseline | 0.0 |
| week 1 | -27.3099 |
| week 2 | -30.7188 |P=0.366
Mean % change of AST (95%CI) (%)
MS
PS
P=0.164
(b) ALT
### Chart
| Category | PS |
|---|---|
| Baseline | 0.0 |
| week 1 | -37.2309 |
| week 2 | -30.1057 |
### Chart
| Category | MS |
|---|---|
| Baseline | 0.0 |
| week 1 | -38.0666 |
| week 2 | -45.0223 |P=0.796
Mean % change of ALT (95%CI) (%)
MS
PS
P=0.138
| MS | 151 | 145 | 137 |
| --- | --- | --- | --- |
| PS | 72 | 59 | 56 |
| MS | 151 | 145 | 137 |
| --- | --- | --- | --- |
| PS | 72 | 59 | 56 |
Patients
observed (n)
Patients
observed (n)

## Slide 3
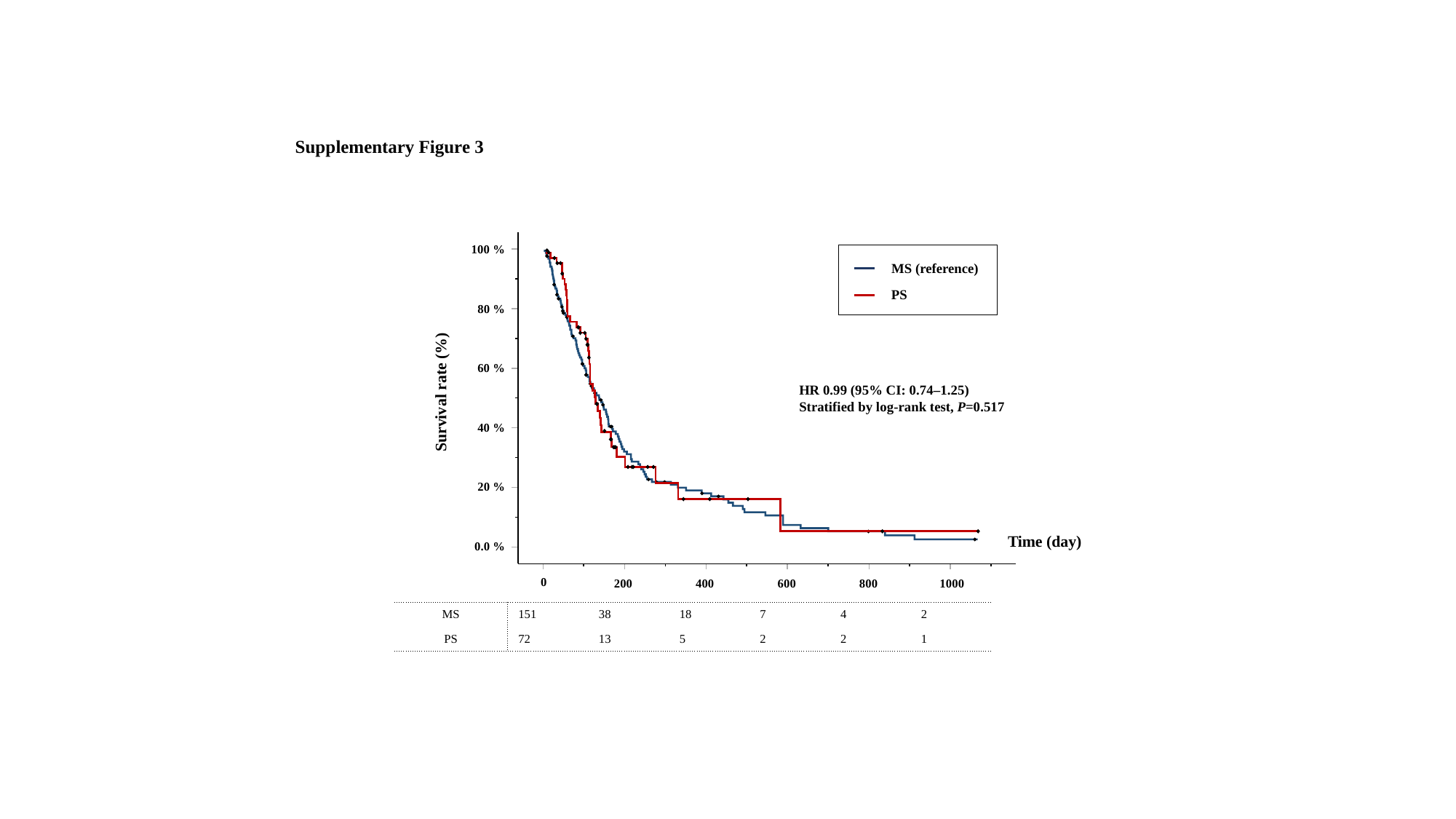

Supplementary Figure 3
100 %
MS (reference)
PS
80 %
60 %
HR 0.99 (95% CI: 0.74–1.25)
Stratified by log-rank test, P=0.517
 Survival rate (%)
40 %
20 %
Time (day)
0.0 %
0
200
400
600
800
1000
| MS | 151 | 38 | 18 | 7 | 4 | 2 |
| --- | --- | --- | --- | --- | --- | --- |
| PS | 72 | 13 | 5 | 2 | 2 | 1 |

## Slide 4
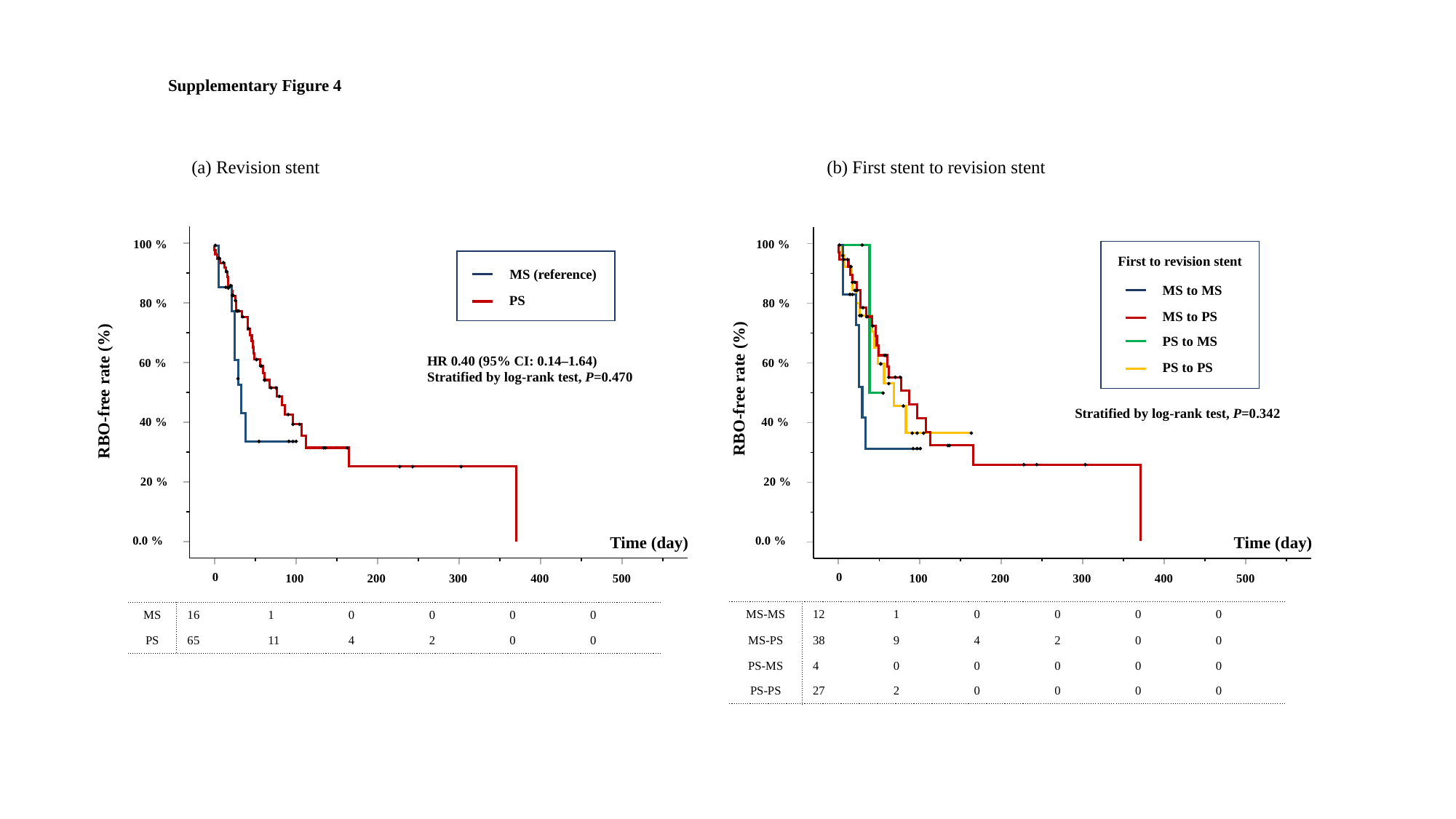

Supplementary Figure 4
(a) Revision stent
(b) First stent to revision stent
100 %
First to revision stent
MS to MS
MS to PS
PS to MS
PS to PS
80 %
60 %
RBO-free rate (%)
Stratified by log-rank test, P=0.342
40 %
20 %
Time (day)
0.0 %
0
100
200
300
400
500
100 %
MS (reference)
PS
80 %
HR 0.40 (95% CI: 0.14–1.64)
Stratified by log-rank test, P=0.470
60 %
RBO-free rate (%)
40 %
20 %
Time (day)
0.0 %
0
100
200
300
400
500
| MS-MS | 12 | 1 | 0 | 0 | 0 | 0 |
| --- | --- | --- | --- | --- | --- | --- |
| MS-PS | 38 | 9 | 4 | 2 | 0 | 0 |
| PS-MS | 4 | 0 | 0 | 0 | 0 | 0 |
| PS-PS | 27 | 2 | 0 | 0 | 0 | 0 |
| MS | 16 | 1 | 0 | 0 | 0 | 0 |
| --- | --- | --- | --- | --- | --- | --- |
| PS | 65 | 11 | 4 | 2 | 0 | 0 |
