# Supplementary material for: Metal stent versus plastic stent in endoscopic ultrasound‐guided hepaticogastrostomy for unresectable malignant biliary obstruction: Large single‐center retrospective comparative study
Source: Dig Endosc. 2024 Nov 15;37(1):117–29. doi: 10.1111/den.14956 (PMC11718138; doi:10.1111/den.14956)
Supplement: Supplementary file 2 — Table S1 Etiology of recurrent biliary obstruction (RBO) until/after the 100 day landmark. Table S2 Technical failure cases of endoscopic re‐intervention. Table S3 Procedures and short‐term outcomes of endoscopic re‐intervention. Table S4 Adjusted relative risk of adverse events in stent types (metal stent vs. plastic stent). [file DEN-37-117-s002.docx]

**Supplementary Table 1. Etiology of RBO until/after the 100-day landmark**

| **Variable** | **All patients**  **n=223** | **MS group**  **n = 151** | **PS group**  **n = 72** | ***P*-value** |
| --- | --- | --- | --- | --- |
| **Early RBO (≤100 days)** | 75 (33.6%) | 46 (30.5%) | 29 (40.3%) | 0.147 |
| - Sludge | 24 (10.8%) | 5 (3.3%) | 19 (26.4%) | <0.001 |
| - Hyperplasia | 14 (6.3%) | 14 (9.3%) | 0 (0.0%) | 0.006 |
| - New biliary stricture | 11(4.9%) | 7 (4.6%) | 4 (5.6%) | 0.500 |
| - Stent dislocation | 12 (5.4%) | 9 (6.0%) | 3 (4.2%) | 0.420 |
| - Stent kinking | 6 (2.7%) | 5 (3.3%) | 1 (1.4%) | 0.369 |
| - Development of stone | 3 (1.4%) | 2 (1.3%) | 1 (1.4%) | 0.692 |
| - Hemobilia | 5 (2.3%) | 4 (2.6%) | 1 (1.4%) | 0.480 |
| **Late RBO (>100 days)** | 23 (10.3%) | 15 (9.9%) | 8 (11.1%) | 0.787 |
| - Sludge | 10 (4.4%) | 4 (2.6%) | 6 (8.3%) | 0.062 |
| - Hyperplasia | 8 (3.6%) | 8 (5.3%) | 0 (0.0%) | 0.042 |
| - New biliary stricture | 2 (0.9%) | 1 (0.6%) | 1 (1.4%) | 0.543 |
| - Development of stone | 3 (1.4%) | 2 (1.3%) | 1 (1.4%) | 0.692 |

Data are presented as n (%).

MS, metal stent; PS, plastic stent; RBO, recurrent biliary obstruction

**Supplementary Table 2. Technical failure cases of endoscopic re-intervention**

|  | **Reason** | **Outcome** | **Number** |
| --- | --- | --- | --- |
| **MS　group** | Difficulty in passing the guidewire due to bile duct hyperplasia | PTCD performed | N=4 |
|  | Difficulty in passing the guidewire due to new biliary stricture | PTCD performed | N=2 |
|  | Difficulty in identifying the fistula due to stent dislocation | New EUS-HGS performed | N=1 |
| **PS group** | Difficulty in retrieving the mislocated stent | ENBD tube placed and retrieved by ballon-catheter through the transpapillary approach in a second session | N=1 |

MS, metal stent; PS, plastic stent; PTCD, percutaneous transhepatic biliary drainage; EUS-HGS, endoscopic ultrasound-guided hepaticogastrostomy; ENBD, endoscopic nasobiliary drainage

**Supplementary Table 3. Procedures and short-term outcomes of endoscopic re-intervention**

|  | **RBO** | | | | |  |
| --- | --- | --- | --- | --- | --- | --- |
| **First stent group** | **MS group**  **n = 50** | |  | **PS group**  **n = 31** | | ***P*-value** |
| Technical success | 50/57 (87.7%) | |  | 31/32 (96.9%) | | 0.143 |
| Clinical success | 45 (90.0%) | |  | 26 (83.9%) | | 0.315 |
| Procedure time, min | 43.0 (30–60) | |  | 45.0 (30.5–60) | | 0.807 |
| Adverse events | 4 (8.0%) | |  | 2 (6.5%) | | 0.581 |
| - Grade (mild/moderate/severe) | 0/3/1 | |  | 0/0/2 | | 0.223 |
| **Revision stent**  **(First stent-revision stent)** | **MS (MS-MS)**  **n = 12** | **PS (MS-PS)**  **n = 38** |  | **MS (PS-MS)**  **n = 4** | **PS (PS-PS)**  **n = 27** |  |
| **Revision stent** |  |  |  |  |  |  |
| Metal stent |  |  |  |  |  |  |
| - Laser-cut type | 7 (58.3%) | - |  | 1 (25.0%) | - | - |
| - Braided type | 5 (41.7%) | - |  | 3 (75.0%) | - | - |
| Plastic stent |  |  |  |  |  |  |
| - Straight type | - | 18 (47.4%) |  | - | 21 (77.8%) | - |
| - Single pigtail type | - | 20 (52.6%) |  | - | 6 (22.2%) | - |
| Clinical success | 8 (66.7%) | 37 (97.4%) |  | 4 (100%) | 22 (81.5%) | 0.021 |
| Procedure time, min | 36.5 (29.5–60) | 44.5 (30–70) |  | 30.5 (18–42.5) | 50 (33.5–60) | 0.173 |
| Adverse events | 1 (8.3%) | 3 (7.9%) |  | 0 (0.0%) | 2 (7.4%) | 0.951 |
| - Grade (mild/moderate/severe) | 0/1/0 | 0/2/1 |  | 0/0/0 | 0/0/2 | 0.712 |
| Antegrade stenting | 0 (0.0%) | 10 (26.3%) |  | 0 (0.0%) | 6 (22.2%) | 0.166 |

RBO, recurrent biliary obstruction; MS, metal stents; PS, plastic stents.

**Supplementary Table 4. Adjusted relative risk of adverse events in stent types (metal stent vs. plastic stent)**

|  | **Overall (n = 223)** | | | |
| --- | --- | --- | --- | --- |
| **Variables** | **Univariate**  **OR (95% CI)** | ***P*-value** | **Multivariate**  **aOR (95% CI)** | ***P*-value** |
| Age (per year) | 0.97 (0.95–1.00) | 0.043 |  |  |
| Sex (male) | 1.56 (0.79–3.08) | 0.196 |  |  |
| Performance status (≥ 2) | 1.30 (0.54–3.09) | 0.560 |  |  |
| Primary disease |  |  |  |  |
| - Pancreatic cancer | 1.15 (0.59–2.23) | 0.690 |  |  |
| - Biliary tract cancer | 0.35 (0.13–0.95) | 0.038 | 0.29 (0.09–1.34) | 0.079 |
| Disease status |  |  |  |  |
| - Local invasion (reference) | 1.0 (reference) | - | 1.0 (reference) | - |
| - Metastasis | 1.13 (0.50–2.54) | 0.775 | 1.10 (0.47–2.56) | 0.822 |
| - Recurrence | 0.44 (0.14–1.41) | 0.168 | 0.43 (0.13–1.44) | 0.170 |
| Stenosis status |  |  |  |  |
| - Hilar stenosis (vs. distal stenosis) | 0.97 (0.45–2.08) | 0.943 | 2.10 (0.83–5.33) | 0.120 |
| Cholangitis status | 1.10 (0.50–2.42) | 0.821 | 1.21 (0.51–2.85) | 0.665 |
| Ascites | 1.42 (0.65–3.10) | 0.373 | 1.53 (0.67–3.50) | 0.314 |
| Re-intervention after ERCP | 0.77 (0.35–1.67) | 0.503 |  |  |
| Previous duodenal stent deployed | 1.13 (0.54–2.38) | 0.752 |  |  |
| Stenting to B2 (vs. B3) | 1.91 (0.95–3.82) | 0.070 |  |  |
| Puncture needle, 22 G (vs. 19 G) | 2.02 (0.66–6.16) | 0.216 |  |  |
| Fistula dilation | 1.36 (0.44–4.17) | 0.593 | 1.47 (0.45–4.78) | 0.521 |
| Stent length (≥ 10 cm) | 0.69 (0.34–1.37) | 0.284 |  |  |
| Simultaneous duodenal stenting | 0.67 (0.22–2.03) | 0.476 |  |  |
| Plastic stents (vs. Metal stents) | 0.34 (0.15–0.82) | 0.016 | 0.32 (0.12–0.83) | 0.019 |
| Timing 2021- (vs. 2018-2020) | 0.80 (0.41–1.55) | 0.501 | 1.26 (0.59–2.68) | 0.556 |
| Non-expert hand (vs. expert hand) | 1.30 (0.70–1.95) | 0.569 | 1.28 (0.54–2.75) | 0.254 |

OR, odds ratio; CI, confidence interval; aOR, adjusted odds ratio; ERCP, endoscopic retrograde cholangiopancreatography; 22 (19) G, 22 (19) gauge; vs., versus.
